# Evaluating myelophil, a 30% ethanol extract of *Astragalus membranaceus* and *Salvia miltiorrhiza*, for alleviating fatigue in long COVID: a real-world observational study

**DOI:** 10.3389/fphar.2024.1394810

**Published:** 2024-06-20

**Authors:** Jin-Yong Joung, Jin-Seok Lee, Yujin Choi, Yoon Jung Kim, Hyeon-Muk Oh, Hyun-Sik Seo, Chang-Gue Son

**Affiliations:** ^1^ Department of Internal Medicine, Daejeon Good-morning Korean Medicine Hospital, Daejeon, Republic of Korea; ^2^ Institute of Bioscience and Integrative Medicine, Daejeon University, Daejeon, Republic of Korea; ^3^ Department of Internal Medicine, Daejeon Korean Medicine Hospital of Daejeon University, Daejeon, Republic of Korea

**Keywords:** Long COVID, fatigue, Myelophil, *Astragalus membranaceus*, *Salvia miltiorrhiza*

## Abstract

**Background:**

Persistent post-infectious symptoms, predominantly fatigue, characterize Long COVID. This study investigated the efficacy of Myelophil (MYP), which contains metabolites extracted from *Astragalus membranaceus* and *Salvia miltiorrhiza* using 30% ethanol, in alleviating fatigue among subjects with Long COVID.

**Methods:**

In this prospective observational study, we enrolled subjects with significant fatigue related to Long COVID, using criteria of scores of 60 or higher on the modified Korean Chalder Fatigue scale (mKCFQ11), or five or higher on the Visual Analog Scale (VAS) for brain fog. Utilizing a single-arm design, participants were orally administered MYP (2,000 mg daily) for 4 weeks. Changes in fatigue severity were assessed using mKCFQ11, Multidimensional Fatigue Inventory (MFI-20), and VAS for fatigue and brain fog. In addition, changes in quality of life using the short form 12 (SF-12) were also assessed along with plasma cortisol levels.

**Results:**

A total of 50 participants (18 males, 32 females) were enrolled; 49 were included in the intention-to-treat analysis with scores of 66.9 ± 11.7 on mKCFQ11 and 6.3 ± 1.5 on the brain fog VAS. After 4 weeks of MYP administration, there were statistically significant improvements in fatigue levels: mKCFQ11 was measured at 34.8 ± 17.1 and brain fog VAS at 3.0 ± 1.9. Additionally, MFI-20 decreased from 64.8 ± 9.8 to 49.3 ± 10.8, fatigue VAS dropped from 7.4 ± 1.0 to 3.4 ± 1.7, SF-12 scores rose from 53.3 ± 14.9 to 78.6 ± 14.3, and plasma cortisol levels also elevated from 138.8 ± 50.1 to 176.9 ± 62.0 /mL. No safety concerns emerged during the trial.

**Conclusion:**

Current findings underline MYP’s potential in managing Long COVID-induced fatigue. However, comprehensive studies remain imperative.

**Clinical Trial Registration:**

https://cris.nih.go.kr, identifier KCT0008948.

## 1 Introduction

Long COVID, often referred to as post-acute sequelae of COVID-19, is a multifaceted condition marked by persistent and frequently severe symptoms that emerge 2–3 months after an infection with the severe acute respiratory syndrome coronavirus 2 (SARS-CoV-2). Common manifestations include fatigue, body pain, mood disturbances, cognitive issues, and respiratory complications (Chuang et al., 2023). Fatigue, often accompanied by cognitive complaints like “brain fog”, is one of the most challenging symptoms of Long COVID (Chasco et al., 2022). The prevalence of post-COVID-19 fatigue ranges from 9% to 58%, influenced by follow-up duration, study population characteristics, recruitment methods, and evaluation depth (Verveen et al., 2022). Given the profound medical and socio-economic implications of Long COVID’s fatigue, particularly its effects on work productivity and quality of life (Lunt et al., 2022), it’s imperative that affected individuals receive specialized care and support.

While treatments for Long COVID fatigue are still emerging, behavioral interventions have shown potential efficacy in addressing post-infection fatigue conditions (Kuut et al., 2023). Currently, there’s no established drug treatment targeting Long COVID fatigue. However, strategies initially designed for Myalgic Encephalomyelitis/Chronic Fatigue Syndrome (ME/CFS), a condition that shares pathophysiological similarities with Long COVID, are under investigation (Qanneta, 2022). Recent studies have particularly highlighted their shared hallmarks in immune dysregulation, energy metabolism, and the pivotal role of the hypothalamic-pituitary-adrenal (HPA) axis (Komaroff and Lipkin, 2023).

One such potential therapeutic agent is Myelophil (MYP), a 1:1 mixture of the 30% ethanol extracts of *Astragalus membranaceus* and *Salvia miltiorrhiza*. Traditionally used to treat chronic fatigue-related disorders, MYP exhibited moderate benefits in a recent phase 2 RCT with 98 ME/CFS patients, showing pronounced effectiveness for those with severe symptoms (Joung et al., 2019a). Its potential benefits for ME/CFS highlight the need to investigate its effectiveness specifically against Long COVID-related fatigue, which may involve unique pathophysiological pathways influenced by SARS-CoV-2.

In this prospective observational study, our objective is to evaluate the effectiveness of MYP in alleviating fatigue symptoms in Long COVID patients. We aim to observe and analyze real-world data from patients who have opted to include MYP in their treatment regime, providing valuable insights into its utility in a practical healthcare setting.

## 2 Materials and methods

### 2.1 Participants

This study aims to analyze the outcomes --in individuals who had recovered from COVID-19 and were experiencing severe fatigue or brain fog symptoms and have opted to use MYP as part of their treatment. The data collection is conducted at Daejeon Korean Medicine Hospital of Daejeon University.

Eligible participants were those aged between 13 and 70 who, following a 4-week recovery period, reported these persistent symptoms. The diagnosis of COVID-19 for these participants was verified using the South Korean government’s public health system, which provided online access to medical records of individuals diagnosed with COVID-19 until 30 May 2023. Polymerase Chain Reaction (PCR) testing to monitor viral loads was not employed as the study focused on individuals beyond the acute phase of infection, confirmed using rapid antigen tests to verify recovery status and align with the study’s aim of assessing MYP’s effects on Long COVID symptoms.

Eligibility for data inclusion requires a score of 60 or higher on the Modified Korean version of the Chalder Fatigue Scale (mKCFQ11), or a score of five or higher on the Visual Analog Scale (VAS) for brain fog. We exclude data from individuals with potential alternative causes for fatigue such as chronic hepatic, cardiovascular, neurological diseases, hypothyroidism, or clinically significant anemia, and those taking other supplements for fatigue/brain fog, with major physical or mental health issues, or recently involved in other clinical trials. For detailed inclusion and exclusion criteria, refer to Supplementary Table S1.

All participants provided informed consent prior to participation. The study was based on the principles of the Declaration of Helsinki and has received ethical clearance from the Institutional Review Board (IRB) of Daejeon University’s Korean Medicine Hospital, with the reference number DJDSKH-21-BM-19. Additionally, the study was registered in the Clinical Research Information Service of the Republic of Korea (KCT0008948).

### 2.2 Study design and treatment

In this prospective observational study, we reference prior clinical trials in ME/CFS where MYP was administered for 4 weeks, providing a basis for our focus on a similar treatment duration for Long COVID symptoms (Joung et al., 2019a). We observed the effects of MYP over this 4-week period in individuals recovering from COVID-19 who report experiencing fatigue and brain fog. During this time, participants are typically advised to consume two MYP capsules orally, twice daily, leading to a total daily dosage of 2,000 mg. According to the Consensus statement on the Phytochemical Characterisation of Medicinal Plant extracts (ConPhyMP) guidlines (Heinrich et al., 2022), MYP, not listed in any country’s pharmacopoeia, is classified as a Type B extract due to its commercial utilization.

The MYP capsules were manufactured by Hankook BioPharm Pharmacy, adhering to Korean Good Manufacturing Practice guidelines. Each capsule contained 500 mg of a dried extract prepared with 30% ethanol. This extract was derived in equal proportions from two botanical sources: 1.389 g each of *A. membranaceus* Fisch. ex Bunge (Fabaceae; *A. membranaceus* radix et rhizoma) and *S. miltiorrhiza* Bunge (Lamiaceae; *Salviae miltiorrhizae* radix et rhizoma). The *A. membranaceus* was sourced from Jecheon, South Korea (Batch No. 20191104-JC-HG), and the *S. miltiorrhiza* came from Hebei, China (Batch No. 20200228-CHN-DS), both purchased from Jeong-Seong Drugstore in Daejeon, Korea. The extraction of MYP involved a 20-h process at 80°C with 30% ethanol, yielding a final product concentration of 20.52% (w/w), which was then stored for subsequent use. The extraction of MYP was performed over a 20-h period at 80°C using 30% ethanol. This process resulted in a final product concentration of 20.52% (w/w), corresponding to a drug-extract ratio of 4.87:1, indicating that approximately 4.87 g of raw material were used to obtain 1 g of extract. Subsequently, the extract was stored for future use. The detailed specifications of MYP are shown in Supplementary Table S2.

**FIGURE 1:**
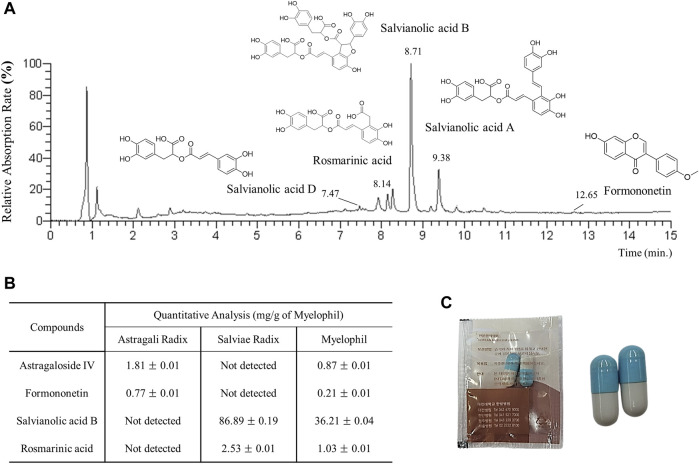
**(A)** UHPLC-MS chromatogram of MYP. **(B)** Quantitative LC-MS analysis of MYP with four reference compounds. **(C)** Image of MYP capsules.

To ensure the consistency of MYP’s components, we performed fingerprint analysis as previously outlined (Kim et al., 2014), using four reference compounds: astragaloside IV and formononetin from *A. membranaceus*, and salvianolic acid B and rosmarinic acid from *S. miltiorrhiza*. For this analysis, 20 mg of MYP and 10 µg of each reference compound were dissolved in 1 mL of 90% methanol and filtered through a 0.45 µm filter. The samples were analyzed with ultra-high-performance liquid chromatography-mass spectrometry (UHPLC-MS) and liquid chromatography-mass spectrometry (LC-MS) using an LTQ Orbitrap XL system equipped with an electrospray ionization source. Chromatographic separation was carried out on an Acquity BEH C18 column using 0.1% formic acid in water (mobile phase A) and 0.1% formic acid in acetonitrile (mobile phase B) at a flow rate of 0.3 mL/min. The elution gradient was programmed to maintain 10% B isocratically for 0–1 min, linearly increase from 10% to 90% B over 1–10 min, and hold at 100% B from 10–12 min, ensuring thorough and consistent analysis of the metabolites. The representative sample chromatogram and the corresponding quantitative analysis are presented in Figures 1A, B. The capsule image is shown in Figure 1C.

### 2.3 Assessment of fatigue and safety

The primary outcome was the change in mKCFQ11 scores after 4 weeks of MYP administration, a tool specifically designed to assess fatigue severity with established reliability and validity (Ahn et al., 2020). The mKCFQ11 comprises 11 questions: seven on physical fatigue (up to 63 points) and four on mental fatigue (up to 36 points), with a combined maximum of 99 points. For secondary measures, the study employed the Multidimensional Fatigue Inventory (MFI-20), the VAS for general fatigue, a specific VAS for brain fog, and the Quality of Life (SF-12) scale to gauge the participants’ overall wellbeing.

To explore MYP’s pharmacological effects, we measured plasma cortisol with an R&D Systems assay kit (cat. No. KGE008B, Minneapolis, United States) and recorded absorbance at 450 and 570 nm using a Molecular Devices spectrophotometer (Sunnyvale, CA, United States) during fasting hours pre- and post-treatment. Furthermore, a complete blood count (CBC), chemistry profile, and urinalysis were performed to ensure the safety of MYP.

### 2.4 Estimation of sample size

Using G*Power software (version 3.1.9.7) (Faul et al., 2007), we estimated the necessary sample size for our study. Given that our study design involves a single-arm pre-post comparison, we used the standardized mean difference (SMD) as our effect size measure. We set the significance level at 0.05, effect size at 0.5, and statistical power at 0.90. An SMD of 0.5, indicating a medium effect size, was chosen based on clinical experience and a recent phase 2 RCT involving MYP, which demonstrated benefits in reducing fatigue among patients with chronic fatigue syndrome (Joung et al., 2019a). This resulted in a minimum required sample size of 44 participants. Anticipating a dropout rate of approximately 10%, we decided to enroll 50 participants to ensure the robustness of our findings. Detailed calculations are provided in the Supplementary Table S3.

### 2.5 Statistical analysis

Analyses were conducted on the intention-to-treat (ITT) population using the baseline observation carried forward (BOCF) method, which included all participants who completed the baseline assessment and received at least one dose of MYP. A safety analysis encompassed all participants who received at least one dose of the trial medication. Continuous measures, including primary and secondary outcomes from mKCFQ11, MFI-20, VAS, SF-12, and cortisol levels, were compared pre- and post-intervention. The normality of the data was assessed using the Shapiro-Wilk test. For data following a Gaussian distribution, paired *t*-tests were employed. For non-Gaussian data, the Wilcoxon Signed-Rank Test was utilized. Additionally, correlation analyses were performed for these indices. A *p*-value of less than 0.05 was considered statistically significant.

## 3 Results

### 3.1 Study population

From December 2021 to April 2023, a total of 50 participants (18 males and 32 females) were enrolled. However, one female participant withdrew due to personal circumstances before the drug administration began. Of the remaining 49 participants (18 males and 31 females) who successfully finished the 4-week treatment and maintained an adherence rate above 75%, all were considered for the ITT analysis.

The mean age of the 49 participants was 42.0 ± 12.2 years (males: 43.3 ± 11.8; females: 41.9 ± 12.2), with a mean BMI of 23.6 ± 3.4 (males: 24.9 ± 2.6; females: 22.9 ± 3.6). On average, participants commenced the trial 139.3 ± 81.4 days after their COVID-19 diagnosis. At baseline, participants displayed pronounced fatigue, with an average mKCFQ11 score of 66.9 ± 11.9 (physical fatigue: 45.1 ± 6.6 and mental fatigue: 21.9 ± 6.6). Their average brain fog VAS score was 6.3 ± 1.5.

### 3.2 Changes in primary assessment: mKCFQ11 score

**FIGURE 2:**
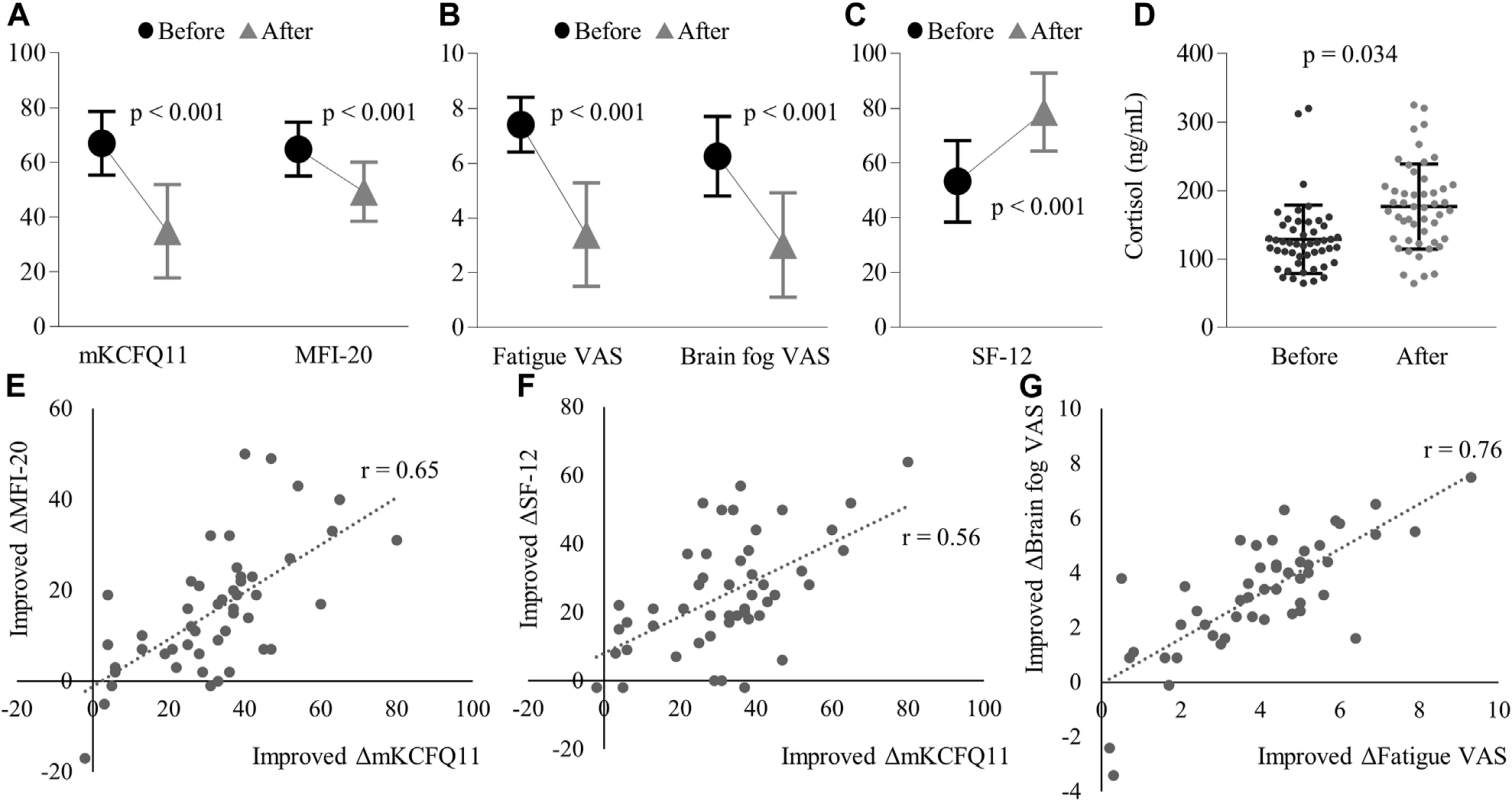
Changes before and after treatment in: **(A)** mKCFQ11 and MFI-20 scores, **(B)** Fatigue VAS and Brain fog VAS scores, **(C)** SF-12 scores, and **(D)** Plasma cortisol levels (ng/mL). The figure also demonstrates the correlations of improved changes between **(E)** mKCFQ11 and MFI-20, **(F)** mKCFQ11 and SF-12, and **(G)** Fatigue VAS and Brain fog VAS. Note: ‘r’ represents the correlation coefficient. Error bars represent standard deviations. Significant reductions were observed in mKCFQ11, MFI-20, Fatigue VAS, Brain Fog VAS, and SF-12 (all *p* < 0.001, data followed Gaussian distribution). Plasma cortisol levels increased significantly post-treatment (*p* = 0.034, non-Gaussian distribution).

After 4 weeks of treatment, the mKCFQ11, our primary assessment, exhibited a significant shift from 66.9 ± 11.7 to 34.8 ± 17.1 (*p* < 0.001). Both components, physical fatigue (from 45.1 ± 6.6 to 23.8 ± 11.3) and mental fatigue (from 21.9 ± 6.6 to 11.1 ± 7.1), showed marked reductions (*p* < 0.001 for both) (Figure 2A). The changes in mKCFQ11 scores were tested for normality using the Shapiro-Wilk test, and the results indicated that the data followed a Gaussian distribution (Shapiro-Wilk statistic = 0.969, *p* = 0.218).

### 3.3 Changes in secondary assessment

Secondary assessments showed statistically significant improvements with *p*-values less than 0.001. MFI-20 shifted from 64.8 ± 9.8 to 49.3 ± 10.8, Fatigue VAS changed from 7.4 ± 1.0 to 3.4 ± 1.9, Brain Fog VAS decreased from 6.3 ± 1.5 to 3.0 ± 1.9, and SF-12 increased from 53.3 ± 14.9 to 78.6 ± 14.3 (Figures 2A–C).

The normality of these changes was also tested using the Shapiro-Wilk test. The results indicated that MFI-20 (Shapiro-Wilk statistic = 0.967, *p* = 0.192), Fatigue VAS (Shapiro-Wilk statistic = 0.981, *p* = 0.592), Brain Fog VAS (Shapiro-Wilk statistic = 0.959, *p* = 0.089), and SF-12 (Shapiro-Wilk statistic = 0.969, *p* = 0.216) followed a Gaussian distribution.

In the correlation analysis, the mKCFQ11 showed strong positive correlations with MFI-20 (correlation coefficient *r* = 0.65), Fatigue VAS (*r* = 0.70), and Brain Fog VAS (*r* = 0.54), while demonstrating a strong negative correlation with SF-12 (*r* = −0.59). (Figures 2E–G).

Additionally, there was a marked rise in cortisol levels post-treatment. The cortisol levels increased from 138.8 ± 50.1 ng/mL pre-treatment to 176.9 ± 62.0 ng/mL post-treatment (*p* < 0.001). (Figure 2D). The changes in cortisol levels did not follow a Gaussian distribution (Shapiro-Wilk statistic = 0.952, *p* = 0.046), so the Wilcoxon Signed-Rank Test was used to analyze these changes. This elevation in cortisol showed no significant correlation with mKCFQ11, with r being 0.15.

#### 3.3.1 Safety

One participant (2.04%) experienced mild indigestion but recovered without any specific treatment. No other adverse reactions, including liver and kidney function in blood tests, were observed (data not shown).

## 4 Discussion

In traditional Korean and Chinese medicine, *A. membranaceus and S. miltiorrhiza* are respectively regarded as fundamental botanical drugs for enhancing two essential components of the human body, Qi and blood, respectively. Qi, understood as the vital energy, sustains bodily operations, including metabolism and growth, whereas blood serves as the crucial nourishing agent. Deficiencies in Qi or blood are linked to symptoms of physical and mental exhaustion (Kim et al., 2016). MYP, as a mixture of these two botanicals, has shown potential in treating ME/CFS.

Preclinical studies have demonstrated that MYP not only protects central neurons from stress-induced damage but also relieves fatigue and cognitive impairment by modulating the HPA axis, inhibiting neuroinflammation, and regulating cholinergic activity (Lee et al., 2015; Song et al., 2021). The optimal dosage for MYP, informed by these studies and further animal toxicity investigations, achieved peak effectiveness in mice at dosages exceeding 200 mg/kg/day (Kim et al., 2014). Additionally, the safe dosage for humans, or the no-observed-adverse-effect level (NOAEL), was established at 694 mg/kg, following toxicity evaluations with both rodents and non-rodents (beagle dog) (Joung et al., 2019b). A phase 2 clinical trial highlighted MYP’s potential, showing notable benefits in treating ME/CFS, especially for individuals with severe symptoms (Joung et al., 2019a).

Recent research into Long COVID has revealed significant disruptions such as T-cell dysregulation, systemic inflammation, and a disjointed immune response to SARS-CoV-2 (Yin et al., 2024). This condition is marked by increased migration of CD4^+^ T-cell to inflamed tissues, exhaustion of SARS-CoV-2-specific CD8^+^ T-cell, and heightened antibody levels, creating a mismatch between cellular and humoral responses. MYP might counteract these issues through its actions on both the central nervous system and systemic inflammation. It modulates neurotransmitter pathways, notably serotonin and dopamine, which are known to alleviate neuroinflammatory processes and neurotransmitter imbalances, issues prevalent in Long COVID (Song et al., 2021; Reiss et al., 2023). Additionally, MYP’s regulation of key mediators like transforming growth factor β (TGF-β) and its influence on the HPA axis provide anti-inflammatory benefits across multiple organ systems, potentially reducing the widespread inflammation characteristic of Long COVID (Kim et al., 2013; Lee et al., 2019). Given these properties, we initiated this real-world observational study to investigate the potential of MYP in alleviating symptoms of Long COVID fatigue.

The radical reduction of fatigue symptoms post-MYP treatment, as reflected in the mKCFQ11 scores (approximately 50% of baseline severity), offers a promising insight into potential interventions for Long COVID-induced fatigue. Additionally, this anti-fatigue efficacy of MYP is strongly supported by other measurements using fatigue-related tools: 24% in MFI-20, 54% in fatigue VAS, and 52% in brain fog VAS (Figures 2A, B). As expected, the QOL level also improved notably, showing a 47% increase from the baseline score of SF-12 (Figure 2C). Furthermore, there are strong and consistent correlations among the changed scores of these measurements (Figures 2E–G). Such a transition underscores a significant overall improvement in fatigue in Long COVID patients following MYP administration. Based on our prior research, the mKCF11 scale scores can be interpreted as follows: 0–25 points suggest no/mild fatigue; 25–40 indicate general fatigue; 40–60 represent idiopathic chronic fatigue levels; and scores exceeding 60 are indicative of ME/CFS levels (Lim and Son, 2022). At the baseline, 40 participants exhibited intense fatigue comparable to ME/CFS levels. However, after 4 weeks of MYP treatment, only four participants still had scores above 60. Remarkably, post-treatment, 35 participants had scores of 40 or below, of which 14 achieved scores of 25 or less (data not shown).

In our real-world observational study, we documented significant effects of MYP on Long COVID-related fatigue, highlighting the importance of patient-centered, value-based outcomes in contemporary medical practice. While our study’s design, a non-randomized, open-label observational study without a control group, calls for a careful interpretation of these results, the observed improvements are nonetheless compelling. When compared to placebo effects reported in prior RCTs, our findings suggest a potentially greater efficacy of MYP. For example, previous RCTs on Long COVID fatigue using the Chalder Fatigue Questionnaire (CFQ-11, with a maximum possible score of 33) or the Visual Analog Fatigue Scale (VAFS, with a maximum possible score of 10) demonstrated varying placebo responses: one exhibited a 5.7% decrease over 4 weeks, reducing from 28.1 to 26.5 on the CFQ-11 (Finnigan et al., 2023), while another reported a 22.5% decline in just 14 days, moving from 25.7 to 20.0 on the CFQ-11 (Rathi et al., 2021), and a further study noted an 18.7% reduction in VAFS scores over 2 weeks, from 7.34 to 5.97 (Harandi et al., 2024, p. 19). In their respective treatments, the metabolic modulator group experienced a 19.9% decrease in CFQ-11 (from 26.2 to 21.0), whereas the enzyme complex and probiotic group achieved a 67.1% improvement in CFQ-11 (from 25.8 to 8.5). Similarly, the Amantadine group demonstrated a 57.3% reduction in VAFS (from 7.90 to 3.37). These comparisons suggest that MYP may offer benefits beyond those attributable to placebo, highlighting the need for further controlled research to validate these promising results.

Given that 55.9% of patients with Long COVID-attributed fatigue reported enduring symptoms for six to 12 months and 17.6% for over a year (Oliveira et al., 2023), there’s a clear persistence of these fatigue symptoms. Strikingly, this enduring fatigue closely resembles the core symptoms of ME/CFS, especially evident in shared manifestations like Brain Fog such as memory and concentration decline (Komaroff and Lipkin, 2023). Such parallels have rekindled interest in the traditional hypothesis associating viral infections with the etiology of ME/CFS (Wong and Weitzer, 2021). The 52% reduction in brain fog VAS in our study suggests that the alleviation of fatigue may be intricately linked to the pharmaceutical activities of MYP on brain pathology. In our previous animal studies, MYP demonstrated notable brain focused effects by modulating neurotransmitter pathways, regulating TGF-β expression, and protecting against chronic cold-stress-induced brain damage in mice (Kim et al., 2013; Song et al., 2021). Based on these findings, we cautiously suggest that improvements related to MYP could offer a potential therapeutic advantage, though not conclusively establishing its dominance.

A noteworthy aspect of our findings was the marked elevation in cortisol levels post-treatment. Cortisol, often termed the “stress hormone”, plays intricate roles in metabolism, immune responses, and the maintenance of circadian rhythms (Russell and Lightman, 2019). Its post-treatment rise, in tandem with the observed reduction in fatigue symptoms, raises an intriguing hypothesis. This pattern could suggest a potential recalibration of the HPA axis, which is frequently dysregulated in chronic fatigue conditions (Tomas et al., 2013). However, the correlation between cortisol levels and fatigue scores was not statistically significant, necessitating cautious interpretation of these initial results. To further understand this relationship and explore MYP’s therapeutic potential for Long COVID fatigue, larger-scale research using double-blind, placebo-controlled studies is essential.

This study has several limitations that are important to consider. Firstly, the absence of a control group in this observational study significantly limits our ability to definitively attribute observed effects to the intervention alone, without potential placebo influences. Secondly, the open-label nature of the study could introduce bias, as participants’ awareness of the treatment might affect their symptom reporting. Thirdly, with a limited participant pool, our results might not be universally applicable, as the demographic may not reflect the diverse spectrum of Long COVID patients. Fourthly, the 4-week timeframe may not sufficiently capture the long-term efficacy or potential side effects of MYP.

Additionally, our study did not control for variables such as participants’ diets or exercise routines, which could influence the effects observed and introduce bias. Nutritional supplements and a balanced diet, recommended for alleviating symptoms of post-COVID-19 fatigue syndrome, may include essential fatty acids, antioxidants, and nutrients like vitamin C, B vitamins, sodium, magnesium, and zinc, which are known to mitigate symptom severity (Barrea et al., 2022). Additionally, physical activity is considered a potential method to alleviate Long-COVID fatigue, although definitive data supporting its effectiveness is currently insufficient (Coscia et al., 2023). Lastly, the absence of assessments for oxygen saturation and detailed pulmonary evaluations is a notable limitation. Pulmonary impairments, often identified through tests like pulmonary function tests, 6-min walk tests, and quality of life assessments, are frequently reported in Long COVID cases (Christopher et al., 2024). Such declines in pulmonary function are crucial contributors to the fatigue, malaise, and decreased quality of life experienced by patients with Long COVID.

Given the prevalent challenges of Long COVID-induced fatigue and the absence of effective treatments, our findings hint at the potential therapeutic role of MYP. However, rigorous and extensive studies are needed to validate its efficacy and mechanism in treating Long COVID fatigue.

## Data Availability

The raw data supporting the conclusion of this article will be made available by the authors, without undue reservation.
