# Supplementary material for: Evaluating myelophil, a 30% ethanol extract of Astragalus membranaceus and Salvia miltiorrhiza, for alleviating fatigue in long COVID: a real-world observational study
Source: Front Pharmacol. 2024 Jun 20;15:1394810. doi: 10.3389/fphar.2024.1394810 (PMC11222562; doi:10.3389/fphar.2024.1394810)
Supplement: Supplementary file 1 [file Table1.DOCX]

| **Supplementary Table 1. Inclusion and Exclusion Criteria** |
| --- |
| **The inclusion criteria are as follows** |
| 1. Participants who are 13 - 70 years old 2. Participants included those who, following a 4-week recovery period from COVID-19, reported significant fatigue or cognitive disturbances, commonly referred to as 'brain fog', that impede their daily functions. Specifically, eligibility was determined by scores exceeding 60 on the mKCFQ11 or a brain fog severity rating surpassing 5 points as indicated by the VAS. 3. Participants who can complete the relevant questionnaires in this trial. 4. Participants who can give informed consent to participate in this trial |
| **The exclusion criteria are as follows** |
| 1. Participants taking medication for conditions/symptoms other than fatigue, excluding those on medication for hypertension, hyperlipidemia, and diabetes. 2. Participants with:    1. Uncontrolled hypertension (SBP ≥ 170 mmHg).    2. Thyroid dysfunction (TSH < 0.27 uIU/mL or TSH > 4.2 uIU/mL).    3. Severe anemia (Hg ≤ 9.0 g/dL).    4. High-grade fatty liver on ultrasonography or liver function abnormalities (AST, ALT, or Total bilirubin levels > 2 times the upper limit of normal).    5. Abnormal kidney function (creatinine levels > 1.2 times the upper limit of normal). 3. Participants diagnosed with or undergoing treatment for cancer, immune diseases, or any other severe known disease. 4. Participants who have had diseases inducing chronic fatigue in the past 6 months, such as hypothyroidism, anemia, psychiatric disorders, etc. 5. Participants who work at night. 6. Participants who consume alcohol more than twice a week. 7. Participants with a BMI below 16.5 or above 30. 8. Participants taking medication or health supplements specifically aimed at treating fatigue and brain fog. 9. Participants who have undergone organ transplantation or are currently taking immunosuppressive medication. 10. Women who are pregnant, lactating, or of childbearing age and have recently tried to conceive. 11. Participants who have participated in another clinical trial within the last month. 12. Participants who cannot understand or follow the overall treatment of this clinical trial due to significant physical or mental impairments, as judged by the researcher. |

COVID-19: Coronavirus Disease 2019; mKCFQ11: Modified Korean Chalder Fatigue scale; VAS: Visual Analog Scale; SBP: Systolic Blood Pressure; BMI: Body Mass Index.

| **Supplementary Table 2. Compositional and Analytical Profile of Myelophil** | | |
| --- | --- | --- |
| Drug Name | Myelophil (combined equal amount of herb 1 and 2) | |
| Formation | 30% Ethanolic extracts (yield 20.52%) | |
| Drug-extract Ratio | 4.87:1 | |
| Ratio of Components | 50 | 50 |
| Composition | Herb 1 | Herb 2 |
| Herbal Name | *Astragalus membranaceus* | *Salvia miltiorrhiza* |
| Full Taxonomic Name | *Astragalus membranaceus* Fisch. ex Bunge (Fabaceae; *Astragalus membranaceus* radix et rhizoma) | *Salvia miltiorrhiza* Bunge (Lamiaceae; *Salviae miltiorrhizae* radix et rhizoma). |
| Parts Used | Dried Roots | Dried Roots |
| Cultivation Location | Jecheon, Republic of Korea | Hebei, China |
| Batch ID | 20191104-JC-HG | 20200228-CHN-DS |
| Fingerprinting Analysis | 1. UHPLC-MS (Thermo Scientific Co., CA, USA)  2. LC-MS (Thermo Scientific Co., CA, USA) | |
| Reference Standard | 1. Rosmarinic acid  2. Salvianolic acid A  3. Salvianolic acid B  4. Salvianolic acid D  5. Formononetin  6. Astragaloside IV | |

| **Supplementary Table 3. Sample Size Calculation** |
| --- |
| To estimate the necessary sample size for our single-arm pre-post study, we used G*Power software (version 3.1.9.7) (Faul et al., 2007). We employed the standardized mean difference (SMD) as our effect size measure. The parameters for the calculation were set as follows:  - Significance level (α): 0.05  - Effect size (SMD, d): 0.5  - Statistical power (1-β): 0.90  Using these parameters, the minimum required sample size (n) was calculated using the following formula:  n = (Zα/2 + Zβ)^2 / SMD^2  where:  - Zα/2 is the critical value of the normal distribution at α/2 (for α = 0.05, Zα/2 ≈ 1.96)  - Zβ is the critical value of the normal distribution at β (for β = 0.10, Zβ ≈ 1.28)  - SMD is the standardized mean difference (effect size, d = 0.5)  Substituting the values:  n = (1.96 + 1.28)^2 / 0.5^2 = (3.24)^2 / 0.25 = 10.4976 / 0.25 = 41.99 ≈ 42  After calculating the sample size, we adjusted for an anticipated dropout rate of 10%. The adjusted sample size (n adjusted) is calculated as follows:  n adjusted = n / (1 - dropout rate)  Substituting the values:  n adjusted = 42 / (1 - 0.10) = 42 / 0.90 ≈ 46.67 ≈ 50  Thus, to ensure the robustness of our findings, we decided to enroll 50 participants. |
